# T2 bright, clinically dim: pheochromocytoma with malignant potential and an atypical clinical presentation

**DOI:** 10.1007/s00261-025-05228-9

**Published:** 2025-11-05

**Authors:** Yudel Tamayo, Jochen Gerstner Saucedo, Roxana López Yazdani, Mohammad Saleh, Beatriz Madrazo

**Affiliations:** 1 Department of Radiology, University of Miami, Coral Gables, USA; 2 Department of Radiology, University of Colorado Anschutz Medical Campus, Aurora, USA; 3 Department of Pathology, University of Miami, Coral Gables, USA

## Abstract

Pheochromocytomas are uncommon adrenal tumors originating from chromaffin cells, with certain cases exhibiting malignant characteristics and behavior. Malignancy is defined by the presence of metastases to sites that normally lack chromaffin tissue; histologic features alone are insufficient for diagnosis. While typically symptomatic, some tumors are discovered incidentally and lack the classic catecholamine-related triad. We present the case of a 52-year-old man with a history of hypertension and hyperlipidemia who presented for routine evaluation and only reported intermittent left lower quadrant pain. On CT and MRI, a 7 cm right adrenal mass with central necrosis was identified; post-contrast MRI showed peripheral enhancement suggesting a hypervascular malignancy. Biochemical testing showed elevated plasma metanephrines and an elevated urinary metanephrine fraction on confirmatory testing. The patient underwent a right adrenalectomy, and histopathological examination revealed pheochromocytoma with angiolymphatic invasion, extra-adrenal extension, a PASS score of 13, and a Ki-67 index of 15–20%, indicating aggressive histologic features consistent with malignant potential. Postoperative recovery was unremarkable and follow-up showed no recurrence. This case underscores the diagnostic value of multimodal imaging in pheochromocytoma, especially when clinical symptoms are not apparent.

## Introduction

Pheochromocytomas are rare neuroendocrine tumors that develop from chromaffin cells in the adrenal medulla, with an incidence of 0.6 per 100,000 person-years [1]. While generally benign, around 10–15% of adrenal pheochromocytomas demonstrate malignant characteristics, indicated by metastases to non-chromaffin locations, with increased rates noted in extra-adrenal paragangliomas [2, 3]. The clinical presentation is highly variable. While the classic triad includes paroxysmal hypertension, headaches, and diaphoresis, many patients are asymptomatic or present with non-specific symptoms, particularly when tumors are discovered incidentally or lack functional activity [1, 4].

Diagnosis involves a combination of biochemical testing and imaging. The assessment of plasma-free or 24-hour urinary metanephrines is the most sensitive and specific diagnostic method [5]. Pheochromocytomas typically present as hypervascular masses on imaging, frequently exhibiting necrosis or hemorrhage, characterized by being T2 bright and having a pronounced contrast enhancement on MRI [6]. Histopathologic evaluation establishes the diagnosis; however, it lacks reliability in predicting malignancy. The presence of local invasion or metastasis is the sole criterion for determining malignant behavior, while scoring systems like Pheochromocytoma of the Adrenal Gland Scaled Score (PASS) and the Ki-67 index assist in risk stratification [2].

We present a case of a pheochromocytoma with malignant potential diagnosed in an asymptomatic patient through imaging and biochemical analysis, with histologic characteristics confirming its aggressive nature.

## Case presentation

A 52-year-old man with a past medical history of long-standing, well-controlled hypertension and mixed hyperlipidemia presented to his primary care physician for a routine annual visit. He reported experiencing intermittent, stabbing pain localized to the left lower quadrant of the abdomen that began a year prior and had progressively intensified, at times severe enough to bring him to his knees. He denied palpitations, diaphoresis, headaches, weight loss, or any prior oncologic history. The patient had no family history of pheochromocytoma, neuroendocrine tumors, or genetic syndromes such as Multiple Endocrine Neoplasia type 2 (MEN2) or Von Hippel–Lindau disease (VHL). There was no history of prior surgeries, chemotherapy, or radiation.

**Fig. 1:**
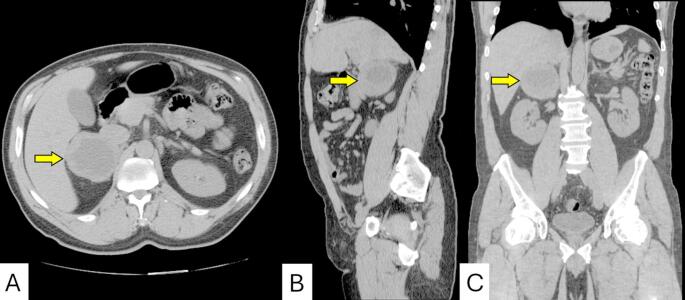
Non-contrast CT of the abdomen in axial (**A**), sagittal (**B**), and coronal (**C**) views demonstrating a large right adrenal mass (yellow arrows). The lesion is well-defined and heterogeneous, measuring approximately 7 cm, with areas of low attenuation suggestive of internal necrosis

**Fig. 2:**
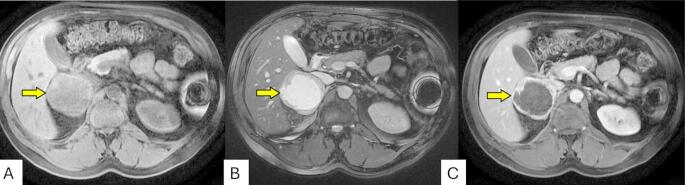
MRI of the abdomen showing a right adrenal mass (yellow arrows) in axial T1-weighted (**A**), T2-weighted (**B**), and post-contrast fat-suppressed T1-weighted (**C**) sequences. **A** T1-weighted image shows an isointense to mildly hypointense right adrenal lesion with well-defined margins. **B** T2-weighted image reveals marked hyperintensity (“light bulb sign”), a characteristic feature of pheochromocytoma. **C** Post-contrast T1-weighted image demonstrates peripheral enhancement with central necrosis

**Fig. 3:**
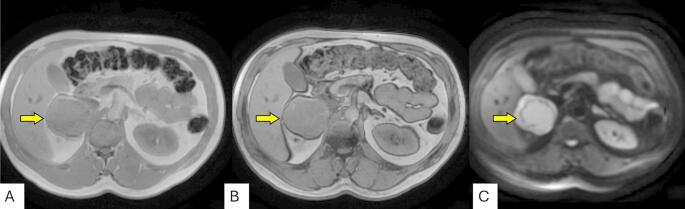
Axial MRI of the abdomen showing a right adrenal mass (yellow arrows) across in-phase (**A**), out-of-phase (**B**), and diffusion-weighted imaging (DWI) sequences (**C**). **A**, **B** The lesion demonstrates no significant signal drop between in-phase and out-of-phase T1-weighted images, suggesting absence of intracellular lipid, which helps exclude lipid-rich adenoma. **C** Diffusion-weighted image shows restricted diffusion within the solid components of the mass, a finding often associated with hypercellularity in malignant pheochromocytoma

Although the pain was localized to the left lower quadrant, physical examination revealed a palpable fullness in the right upper quadrant, prompting further imaging. The discordance between symptom location and mass laterality suggested the abdominal pain may have been unrelated or referred. A computed tomography (CT) scan of the abdomen and pelvis revealed a 6.7 cm solid right adrenal mass with heterogeneous attenuation and central low-attenuation areas suggestive of necrosis (Fig. 1). The mass demonstrated concerning features for a primary adrenal malignancy or metastasis. The left adrenal gland and other abdominal organs were unremarkable. Subsequent abdominal MRI confirmed a 7.1 × 6.8 × 6.2 cm heterogeneous right adrenal lesion (Fig. 2). The lesion was hyperintense on T2-weighted images, demonstrating the classic “light bulb” sign commonly associated with pheochromocytomas, and isointense on T1-weighted sequences. Post-contrast imaging showed peripheral and nodular enhancement with central areas of necrosis. Chemical shift imaging showed no signal loss between in-phase and out-of-phase sequences, helping to exclude lipid-rich adenoma, while diffusion-weighted imaging revealed restricted diffusion in the solid components of the mass, findings supportive of hypercellularity, typical of pheochromocytoma and associated with aggressive behavior (Fig. 3). There was no evidence of vascular invasion or distant metastasis.

Biochemical workup revealed markedly elevated plasma metanephrines (166.1 pg/mL; normal 0–88), prompting suspicion of pheochromocytoma. A 24-hour urinary metanephrine test, performed one month later as confirmatory testing per Endocrine Society guidelines [7], showed a normal total metanephrine level (589 mcg/24 h; reference 224–832) but an elevated metanephrine fraction (246 mcg/24 h; reference 90–315), supporting ongoing catecholamine secretion. The diagnosis was driven by the highly sensitive plasma metanephrines, with the urinary fraction providing complementary specificity [7].

**Fig. 4:**
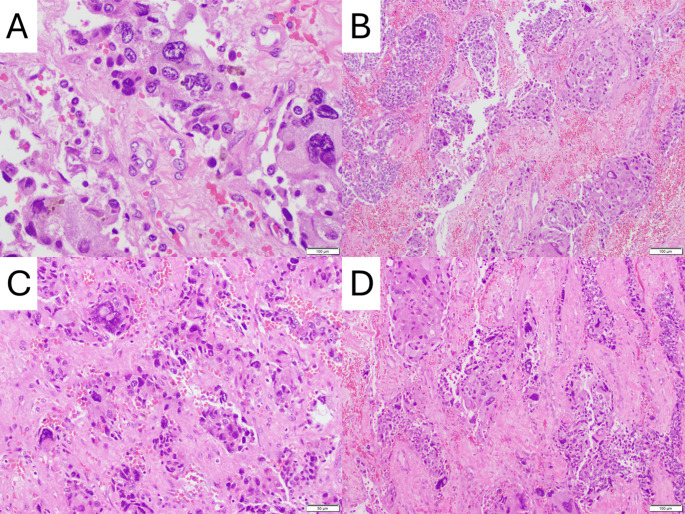
Histopathologic features of the resected pheochromocytoma. **A** High-power view (hematoxylin and eosin [H&E], 100 μm scale) demonstrating marked nuclear pleomorphism, hyperchromasia, and prominent nucleoli within tumor cells, with scattered mitotic figures. **B** Medium-power view (H&E, 100 μm scale) showing classic zellballen architecture composed of nests of polygonal tumor cells separated by vascular stroma, with areas of hemorrhage and necrosis. **C** Intermediate magnification (H&E, 50 μm scale) highlighting hypercellularity, dense fibrous stroma, and focal necrotic debris. **D** Medium-power view (H&E, 100 μm scale) revealing angiolymphatic invasion and trabecular growth pattern, features associated with increased malignant potential

He subsequently underwent right adrenalectomy. Pathologic examination confirmed a 7.0 cm pheochromocytoma with angiolymphatic invasion and extra-adrenal extension. Resection margins were negative. The tumor showed a high proliferative index (Ki-67: 15–20%) and a PASS score of 13, indicating aggressive histologic features associated with malignant potential. Immunohistochemistry was positive for synaptophysin, chromogranin, and S100 (highlighting sustentacular cells), and negative for markers of adrenal cortical or metastatic origin (Fig. 4).

Postoperative recovery was uncomplicated. At six-month follow-up, the patient had gained weight and reported improved well-being. Biochemical remission was supported by serial postoperative plasma metanephrine levels within normal ranges, measured at three, six, and nine months (31, 40, and 39 pg/mL, respectively).

## Discussion

This case illustrates several important aspects of pheochromocytoma evaluation, particularly the role of imaging in detecting malignant behavior in patients lacking classic symptoms. While most pheochromocytomas are discovered due to catecholamine-related crises, an increasing proportion are diagnosed incidentally on imaging, as in this patient [8]. Notably, despite elevated plasma metanephrines, the patient did not present the characteristic triad of pheochromocytomas (headaches, palpitations, and diaphoresis). He instead exhibited severe, localized abdominal pain, an atypical symptom that underscores the extensive and occasionally deceptive clinical spectrum of pheochromocytoma [9, 10]. While the patient’s primary complaint was sharp, episodic pain localized to the left lower quadrant, this did not anatomically correlate with the right adrenal mass identified on imaging. This discrepancy indicates that the presenting pain may have been either unrelated or referred, and that the tumor was discovered incidentally during workup. An alternative explanation is intermittent catecholamine-mediated mesenteric vasoconstriction causing bowel ischemia–type pain; however, this was not demonstrated in our evaluation.

Despite being biochemically active, the tumor did not produce the classic triad of headache, palpitations, and diaphoresis. Several explanations have been proposed for this discordance. Long-standing, sustained hypertension may blunt the hemodynamic effects of catecholamine surges, thereby masking paroxysmal crises. In this patient, the hypertension was long-standing and well-controlled, most consistent with essential hypertension rather than tumor-driven activity. Importantly, pheochromocytomas can manifest with highly variable hypertensive patterns, ranging from sustained hypertension that resembles essential hypertension to paroxysmal episodes triggered by catecholamine surges, and, in some cases, hypertensive crises that may be life-threatening [1, 4]. Additionally, pheochromocytomas may secrete variable proportions of catecholamines or their metabolites, including dopamine, which often produces fewer adrenergic symptoms compared with norepinephrine or epinephrine secretion. Individual differences in adrenergic receptor sensitivity may also modulate clinical expression. These factors highlight the importance of biochemical and imaging evaluation, as reliance on clinical features alone may delay diagnosis.

The biochemical evaluation began with plasma-free metanephrines, which were markedly elevated, thereby guiding the diagnosis of pheochromocytoma. A subsequent 24-hour urine collection, obtained as confirmatory testing in accordance with Endocrine Society guidelines, showed a total metanephrine level within the reference range but an elevated metanephrine fraction, supporting ongoing catecholamine secretion. This apparent discrepancy can be explained by the higher sensitivity of plasma testing compared with urine, while urinary fractionated metanephrines serve as a complementary tool to increase specificity and reduce false positives. Taken together, the elevated plasma free metanephrines established the diagnosis, with the abnormal urinary fraction providing supportive confirmation [7].

The imaging findings in this case (central necrosis, peripheral enhancement, and marked T2 hyperintensity on MRI) are indicative of pheochromocytomas; however, these characteristics can also be suggestive of other malignancies, particularly adrenal cortical carcinomas (ACC) or metastasis [6, 8]. The radiologic differential diagnosis for large adrenal masses with necrosis and heterogeneous enhancement is extensive. ACC generally presents as a large, irregular, heterogeneous mass characterized by internal necrosis, calcification, and hemorrhage, showing high attenuation on unenhanced CT (> 10 Hounsfield units, HU), hyperintensity on T2-weighted MRI, and gradual contrast washout [8, 11, 12]. Metastatic disease, most often from lung, breast, kidney, or melanoma, may be bilateral or unilateral and often shares these imaging features and high fluorodeoxyglucose (FDG) uptake on positron emission tomography/computed tomography (PET/CT) [8, 11, 12]. Lipid-poor adenomas may also mimic malignancy when they lack signal drop on out-of-phase MRI; however, rapid contrast washout remains a distinguishing feature [8, 13]. Myelolipomas are characterized by macroscopic fat on CT and MRI, which differentiates them from other heterogeneous adrenal masses [12, 14]. Other rare mimics include ganglioneuroma, schwannoma, adrenal hemorrhage, and primary adrenal lymphoma [8, 12].

Functional imaging can further refine the differential diagnosis. Metaiodobenzylguanidine (123I-MIBG) scintigraphy has high specificity for pheochromocytoma, 68Ga-DOTA–octreotate (DOTATATE) PET/CT is useful for neuroendocrine tumors, and 18 F-FDG PET/CT reveals high uptake in both malignant and occasionally in benign lesions, though without specificity [6, 15]. Given the significant overlap in cross-sectional imaging findings, a combined approach, integrating lesion size, margins, attenuation, contrast washout, and functional imaging, is essential to guide the diagnosis and treatment planning in its early stages [8, 16].

Histopathologic analysis confirmed aggressive local features (angiolymphatic invasion, extra-adrenal extension, elevated PASS score, and high Ki-67 index) associated with malignant potential. However, as malignancy in pheochromocytoma is strictly defined by the presence of metastases, this case is more accurately described as a pheochromocytoma with malignant potential rather than an established malignant pheochromocytoma [2]. The proliferative index has proven to be a valuable tool in risk stratification, complementing clinical and radiologic evaluations, while surgical resection is the primary treatment for localized malignant pheochromocytoma, proving curative in this instance [1]. While our patient remains disease-free at nine months, this follow-up period is short for a tumor with malignant potential. Current guidelines recommend at least 10 years of annual biochemical testing and periodic imaging, and lifelong surveillance is often appropriate, given the risk of late recurrence or metastasis, particularly when high-risk histologic features are present [1, 5]. Emphasizing structured long-term follow-up is critical, particularly in patients with high-risk histologic features such as extra-adrenal extension, angiolymphatic invasion, or elevated proliferative index [5, 6].

This case highlights the evolving understanding of pheochromocytoma as a disease that can deviate significantly from textbook descriptions, biochemically active but clinically silent, radiologically classic yet histologically aggressive. It emphasizes the vital importance of imaging in the early detection and risk assessment of adrenal neoplasms and reflects the diagnostic and therapeutic difficulties that are becoming more prevalent in modern practice.

## Conclusion

This case demonstrates the significance of including pheochromocytoma in the differential diagnosis of massive adrenal tumors, even in the absence of characteristic symptoms. Multimodal imaging played a key role in identifying malignant features, leading to a prompt surgical intervention confirming the diagnosis of pheochromocytoma with aggressive histologic features consistent with malignant potential. Given the possibility of recurrence, radiologists should be aware of the imaging characteristics that set pheochromocytoma apart from other adrenal lesions.

## Data Availability

No datasets were generated or analysed during the current study.
